# Semaphorin 4D Promotes Skeletal Metastasis in Breast Cancer

**DOI:** 10.1371/journal.pone.0150151

**Published:** 2016-02-24

**Authors:** Ying-Hua Yang, Asma Buhamrah, Abraham Schneider, Yi-Ling Lin, Hua Zhou, Amr Bugshan, John R. Basile

**Affiliations:** 1 Department of Oncology and Diagnostic Sciences, University of Maryland Dental School, Baltimore, Maryland, United States of America; 2 Greenebaum Cancer Center, Baltimore, Maryland, United States of America; 3 Division of Diagnostic and Surgical Sciences, School of Dentistry, University of California Los Angeles, Los Angeles, California, United States of America; University of Texas Southwestern Medical Center, UNITED STATES

## Abstract

Bone density is controlled by interactions between osteoclasts, which resorb bone, and osteoblasts, which deposit it. The semaphorins and their receptors, the plexins, originally shown to function in the immune system and to provide chemotactic cues for axon guidance, are now known to play a role in this process as well. Emerging data have identified Semaphorin 4D (Sema4D) as a product of osteoclasts acting through its receptor Plexin-B1 on osteoblasts to inhibit their function, tipping the balance of bone homeostasis in favor of resorption. Breast cancers and other epithelial malignancies overexpress Sema4D, so we theorized that tumor cells could be exploiting this pathway to establish lytic skeletal metastases. Here, we use measurements of osteoblast and osteoclast differentiation and function *in vitro* and a mouse model of skeletal metastasis to demonstrate that both soluble Sema4D and protein produced by the breast cancer cell line MDA-MB-231 inhibits differentiation of MC3T3 cells, an osteoblast cell line, and their ability to form mineralized tissues, while Sema4D-mediated induction of IL-8 and LIX/CXCL5, the murine homologue of IL-8, increases osteoclast numbers and activity. We also observe a decrease in the number of bone metastases in mice injected with MDA-MB-231 cells when Sema4D is silenced by RNA interference. These results are significant because treatments directed at suppression of skeletal metastases in bone-homing malignancies usually work by arresting bone remodeling, potentially leading to skeletal fragility, a significant problem in patient management. Targeting Sema4D in these cancers would not affect bone remodeling and therefore could elicit an improved therapeutic result without the debilitating side effects.

## Introduction

The semaphorins and their receptors, the plexins, represent a large family of phylogenetically conserved molecules, both secreted and membrane bound, containing a large, cysteine-rich sema domain that were originally identified based on their ability to provide both attractive and repulsive axon guidance cues during neural development. Plexin-semaphorin interactions now have been implicated in a host of responses, including loss of cell-cell contact and branching morphogenesis in epithelium, alterations in cell adhesion, regulation of lymphocyte activation, and development of the heart and vasculature [1]. There are more than 30 semaphorins identified to date classified into 8 subgroups based on their species of origin and sequence similarity, and nine known plexins, Plexin-A1 to -A4, -B1 to -B3, -C1, and -D1, grouped according to domain structure, that exhibit promiscuity in binding and generate varying effects depending on availability and tissue and cell type. Semaphorin 4D (Sema4D) is an immune semaphorin expressed by T-lymphocytes and eosinophils and to a lesser extent by dendritic cells and B-lymphocytes but is also found in nerve tissues, the gonads, kidney, heart, lung and elsewhere, though it has not been found in normal breast epithelium or breast stroma [2]. Our lab and others have shown that it is pro-angiogenic when acting through its receptor, Plexin-B1 on endothelial cells [3,4], and may be produced by malignancies in hypoxia or through aberrant expression of hypoxia inducible factor-1 for the purposes of promoting blood vessel growth into a tumor in a manner analogous to vascular endothelial growth factor [5].

Remodeling of the adult skeleton is controlled by osteoclasts, which resorb bone, and osteoblasts, which deposit bone matrix, and the factors they produce and that are released by the extracellular matrix that mediate this process. Recent studies have identified Sema4D in the bone microenvironment and demonstrate that it is a product of osteoclasts that acts through Plexin-B1 on osteoblasts to inhibit their differentiation and motility [6]. Indeed, Sema4D knockout mice exhibit an osteosclerotic phenotype [6], so this signaling pathway may represent a potential target in the treatment of osteoporosis. However, because excess Sema4D could tip the balance of bone homeostasis in favor of resorption, and we have observed that many carcinomas overexpress Sema4D [7], we wanted to examine if tumor cells could be using this pathway to establish lytic metastatic lesions in the skeleton. It is known that in bone loss associated with neoplasia, a decrease in bone formation is observed in addition to enhanced tumor and osteoclastic resorption [8]. For example, breast cancer cells secrete factors that inhibit osteoblast differentiation and activity [9], influencing their ability to establish lytic skeletal metastases and resulting in pathologic fractures, hypercalcemia and other skeletal related events, a known complication of this disease and other bone-homing malignancies.

Here we show that breast cancer cell lines express high levels of Sema4D relative to their ability to spread to bone and that both soluble (s)Sema4D and Sema4D derived from MDA-MB-231 cells inhibits differentiation and ability to form mineralized matrix by MC3T3 cells, an osteoblast pre-cursor cell line. Our group has shown previously that Sema4D induces production of IL-8 in treated cells [10]. We demonstrate that human and mouse osteoblasts produce IL-8 and lipopolysaccharide-induced CXC (LIX)/CXCL5, the murine homologue of IL-8, respectively, in response to Sema4D, which alone or in combination with RANK-L activates osteoclasts [11], further stimulating bone resorption. Finally, we observed suppression of skeletal spread in a mouse model of metastasis, and increased osteoblasts and decreased numbers of osteoclasts in lesional tissues comprised of MDA-MB-231 cells with silenced Sema4D compared to controls.

Mortality due to cancer is usually related to distant metastasis, so many therapies are directed against this aspect of tumor spread. In bone-homing malignancies, bisphosphonates are used to suppress skeletal metastases, but this form of treatment inhibits osteoclasts and arrests bone remodeling, leading to skeletal fragility and osteonecrosis of the jaw. The results presented here suggest that targeting Sema4D for inhibition could be a viable alternative, since it would not greatly affect skeletal remodeling and therefore could elicit an improved therapeutic result without the debilitating side effects.

## Materials and Methods

### Cell culture

MC3T3-E1 cells (ATCC, Manassas, VA) were maintained in Alpha Minimum Essential Medium (Gibco); 293T (ATCC) and breast cancer cell lines (ATCC) were cultured in DMEM (Sigma, St. Louis, MO); Murine macrophage cell line Raw 264.7 were cultured in RPMI 1640 (Sigma). All media were supplemented with 10% fetal bovine serum (unless otherwise noted) and 100 units/ml penicillin/streptomycin (Sigma) and treated as indicated. Human Osteoblasts (HOB) and osteoblast growth medium were obtained from PromoCell (Heidelberg, Germany).

### Purification of soluble Sema4D

Soluble Sema4D (sSema4D) was produced and purified as described previously [3]. Briefly, the extracellular portion of Sema4D was subjected to PCR and the resulting product cloned into the plasmid pSecTag2B (Invitrogen, Carlsbad, CA). This construct was transfected into 293T cells growing in serum free media. Media containing sSema4D was collected 65 h post-transfection and purified with TALON metal affinity resin (Clontech Laboratories, Palo Alto, CA) according to manufacturer’s instructions. Concentration and purity of the TALON eluates was determined by SDS PAGE analysis followed by silver staining (Amersham Life Science, Piscataway, NJ), comparing band intensity against a BSA standard curve on the same gel, and the Bio-Rad protein assay (Bio-Rad, Hercules, CA). In all cases, media collected from cells transfected with the empty pSecTag2B vector were used as control.

### Alkaline phosphatase assay

Differentiation of MC3T3-E1 cells growing in increasing concentrations of sSema4D was determined by alkaline phosphatase (AP) activity as previously described [12]. Results are expressed as the average and standard deviation for three independent experiments.

### Mineralization assay

Mineralization of MC3T3-E1 cells was determined by Alizarin red (Sigma) in 6-well plate as previously described [13,14]. Briefly, cells were grown to confluency in α-MEM containing 10% fetal bovine serum and 100 units/ml penicillin/streptomycin with osteogenic supplements (50 μM ascorbic acid, 10 nM dexamethasone and 10 mM β-Glycerophosphate) with or without sSema4D or media conditioned by the breast cancer cell lines MDA-MB-231 and MCF-12A and 5 μg/ ml anti-Sema4D antibody (Vaccinex), where indicated. Cultures were incubated at 37°C with 5% CO_2_ with changes of medium every 3 days for 3 weeks. Then, the monolayers were washed with PBS and fixed in 4% paraformaldehyde at room temperature for 15 min. and stained with Alizarin red. For quantitation, photos were taken and stained monolayers were detached from the plate using acetic acid and then transferred to a 1.5 ml Eppendorf tube. The slurry was heated to 85°C for 10 min., and put on ice for 5 min. The supernatant was transferred to a 95-well plate and read in triplicate at 405 nm in a microplate reader.

### Immunoblots and detection of shed Sema4D

The indicated cell lines were grown in serum free media, lysed in buffer (50 mM Tris-HCl, 150 mM NaCl, 1% NP 40 supplemented with protease inhibitors (0.5 mM phenylmethylsulfonyl fluoride, 1 μl/ml aprotinin and leupeptin, Sigma) and phosphatase inhibitors (2 mM NaF and 0.5 mM sodium orthovanadate, Sigma)) for 15 min. at 4°C. After centrifugation, protein concentrations were measured using the Bio-Rad protein assay (Bio-Rad). 100 μg of protein from each sample was subjected to SDS-polyacrylamide gel electrophoresis and transferred onto a PVDF membrane (Immobilon P, Millipore Corp., Billerica, MA). The membranes were then incubated with anti-Sema4D antibody (BD Transduction Labs, BD Biosciences, Palo Alto, CA), anti-Plexin-B1 antibody (Santa Cruz Biotechnology, Santa Cruz, CA) or GAPDH (Sigma). Proteins were detected using the ECL chemiluminescence system (Pierce, Rockford, IL). For detection of shed Sema4D, 4.5 ml of serum free media was placed on MDA-MB-231 or MCF-12A cells, controls or with silenced or overexpressed Sema4D, respectively, growing in 10 cm dishes and left overnight to concentrate protein released into the media. Sample buffer was added directly to the media collected the following day and 50 μl loaded for gel electrophoresis, with protein detected by silver stain (Amersham), as previously described [15].

### IL-8 and LIX/CXCL5 ELISA

ELISA was performed on HOB and MC3T3 cells as previously described [10]. Briefly, confluent HOB and MC3T3 were serum starved for 4 h, then cultured in serum free medium with increasing concentrations of sSema4D. Conditioned media were collected at the indicated time points and used to analyze IL-8 and LIX/CXCL5 production by ELISA by the Cytokine Core Facility, University of Maryland School of Medicine. Where indicated, the assay was performed in the presence of media conditioned by serum starved MDA-MB-231 cells with or without 6 μg/ml C3 toxin (List Biological Laboratories, Campbell, CA). Results are expressed as the average and standard deviation for three independent experiments.

### Osteoclastogenesis assay

2x10^5^ pre-osteoblast RAW 264.7 cells were cultured in each well of a 12-well plate along with RANKL, IL-8, or medium conditioned by HOB treated with 400 ng/ml sSema4D or empty vector transfected controls and 10 μg/ml anti-IL-8 antibody (Lifespan Biosciences), where indicated, for 6 days with media changed every other day. The cells were fixed and tartrate-resistant acid phosphatase (TRAP) staining was conducted using an acid phosphatase, leukocyte (TRAP) kit (Sigma) as previously described [16]. Multinucleated TRAP positive cells were counted as mature osteoclasts in microscopic high power fields. Results are expressed as the average and standard deviation for three independent experiments.

### Short hairpin (sh) RNA, overexpression, and lentivirus infections

The shRNA sequences for human Sema4D and murine Plexin-B1 were obtained from Cold Spring Harbor Laboratory's RNAi library (RNAi Central, http://cancan.cshl.edu/RNAi_central/RNAi.cgi?type=shRNA) [17,18]. Four sequences were used as PCR templates, with the one best silencing the gene of interest chosen for the experiments, as previously reported [7]. Oligos were synthesized (Invitrogen) and cloned into pWPI GW, a Gateway compatible CSCG based lentiviral destination vector as previously described [7,19,20]. Viruses coding for scrambled sequences were used as controls for all lentivirus infections. Viral stocks were prepared and infections performed as previously reported [7]. Cells were checked for viability following lentiviral infections and knockdown was confirmed by immunoblot. For Sema4D overexpression, the extracellular portion of Sema4D was cloned into pSHAG MAGIC2, an entry vector for the Gateway cloning system, and then an LR reaction performed to transfer the inserts into pWPI GW (Invitrogen), as previously described [15].

### Tumor xenografts and bioluminescence imaging

To minimize animal suffering and distress during the grafting procedure, mice were anesthetized with i.p. administration of Avertin anesthesia. An injection site was prepared with an antiseptic povidone iodine scrub and alcohol rinse and 1 x 10^5^ MDA-MB-231 cells, infected *ex vivo* with control virus or lentivirus coding for Sema4D shRNA, were injected into the left cardiac ventricle with a sterile tuberculin syringe with a 25 gauge needle (5/8”) as previously reported [21]. A power analysis based upon published results indicated that 10 mice were required per experimental condition but 11 were used because of an approximately 10% mortality rate for intracardiac injections. However, no adverse effects or animal deaths occurred during grafting. For bioluminescence imaging (BLI), mice were temporarily anesthetized using 2.0–2.5% veterinarian grade isoflurane administered in a dedicated XGI-8 gas inhalation anesthesia apparatus. Once sedated, ocular protectant was applied every 30 minutes, with depth of anesthesia determined by monitoring respiration rate and responsiveness to toe pinch stimuli. Intraperitoneal injections of 100 mg/ml D-luciferin (Xenogen) in PBS were administered to track development of bone metastasis. Images were acquired with a Xenogen IVIS 200 imaging system. For analysis, total photon flux (photons per second) was measured from tumor areas located in the hind limb joints [22,23]. Metastatic lesions formed in control populations 2 to 3 weeks after introduction of tumor cells and were allowed to grow to a measureable size amenable for histopathological analysis (approximately 5 weeks). Mice were monitored every three days as well as on days of bioluminescence (which occurred once a week) for body weight, activity level, ambulation, posture and general overall appearance. The endpoint criteria for euthanizing mice were as follows: any tumor growth that interfered with normal ambulation or sleeping; the inability to drink or eat food; a loss of 20% or more of body weight; tumors causing a mouse to exhibit hydrothorax or hydro-abdomen development to the point of respiratory compromise; a moribund state as indicated by lack of movement to light tapping; mice showing saturating luminescence signals greater than 10^9^ photons/sec/cm^2^ for tumor deposits. Euthanasia was carried out using CO_2_ inhalation followed by cervical dislocation, after which tumors were removed for further sectioning and processing (see below). All animal studies were approved by the University of Maryland Office of Animal Welfare, Institutional Animal Care and Use Committee, in accordance with the NIH Guide for the Care and Use of Laboratory Animals.

### Radiographs

Osteolysis was assessed by X-ray analysis. High resolution digital radiographs (30 kV, 10 s) of hind limbs were performed on anesthetized mice with a Faxitron Digital X-ray system (Faxitron X-Ray, Lincolnshire, IL) as previously described [24], with osteolytic lesions expressed as a percentage of the total tissue area measured using NIH Image J software.

### Serum and supernatant biochemistry

The levels of RANKL, OPG, LIX/CXCL5, IL-8 and osteocalcin (OCN) in the serum and conditioned medium of cultured MC3T3 and HOB cells were determined by ELISA (R&D Systems, Minneapolis, MN, and Immutopic International, San Clemente, CA) as previously described [10]. AP activity was assayed using the SensoLyte pNPP alkaline phosphatase assay kit (AnaSpec, Fremont, CA). Results are expressed as the average and standard deviation for three independent experiments.

### Immunohistochemistry and bone histomorphometric analysis

Hind limb bones were harvested from mice and fixed in 4% paraformaldehyde, decalcified in 0.5M EDTA for 2 weeks, and embedded in paraffin. H&E and TRAP stain (with counterstain) was used for identification of osteoclasts and osteoblasts, and an estimate of the number of osteoblasts per trabecular and cortical area (number/mm^2^) was performed based upon methods previously described [25]. Bone histomorphometry was performed on tumor-bearing hind limb sections using the Osteomeasure bone histomorphometry system (Osteometrix, Atlanta, GA) [22,26]. Analysis of bone volume versus total tissue volume, the number of osteoclasts per bone surface, and the number of osteoblasts per bone surface was assessed according to the recommended nomenclature of the American Society for Bone and Mineral Research [27]. Immunochemical analysis for Sema4D was performed as previously reported [7]. Image acquisition was performed on a Nikon Eclipse E800 Microscope (Nikon, Melville, NY) using 10x and 40x objective lenses.

### Statistical Analysis

Student’s paired *t* tests were performed on means, and *p* values calculated: *, *p* ≤ 0.05; **, *p* ≤ 0.01.

## Results

### Sema4D inhibits osteoblasts in a Plexin-B1-dependent manner

**Fig 1:**
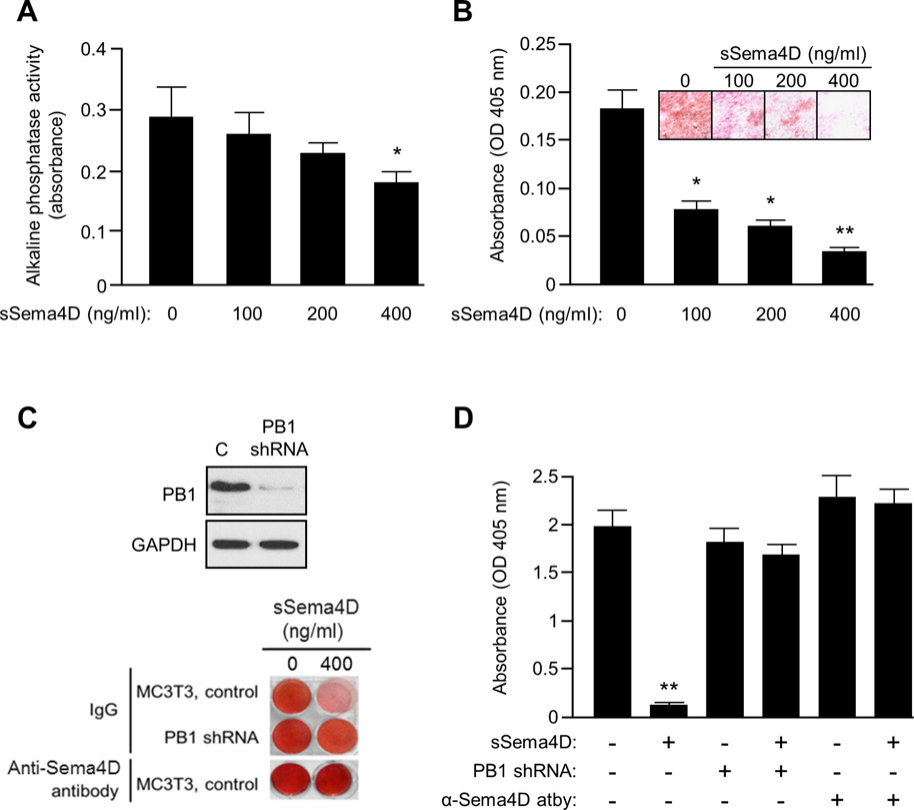
Sema4D inhibits osteoblast differentiation and function in a Plexin-B1-dependent manner. **A.** Alkaline phosphatase activity (Y-axis), a marker of osteoblastic differentiation, decreases in osteoblasts in increasing concentrations of soluble (s)Sema4D. **B.** Absorbance of Alizarin Red (optical density (OD) at 405 nm, Y-axis), which stains mineralized substrate (representative photos shown in the inset), decreases in increasing concentrations of sSema4D for cells growing under differentiating conditions. **C.** MC3T3-mediated matrix deposition and mineralization is inhibited by sSema4D. When Plexin-B1 is silenced with short hairpin (sh) RNA (PB1 shRNA, second row, knockdown confirmed by immunoblot, top), or anti-Sema4D blocking antibody is administered (bottom row), mineralization in the presence of sSema4D is restored. Isotype matched IgG was used as a control (top rows). **D.** Quantification of results by absorbance at 405 nm (Y-axis) of the results shown in C. For all bar graphs *, p ≤ 0.05; **, p ≤ 0.01.

It has been previously shown that Sema4D, originally identified as an immunomodulator and an axon guidance molecule, is produced by osteoclasts and can bind to its receptor Plexin-B1 on osteoblasts to inhibit their motility and ability to produce osteoid and, subsequently, new bone [6]. To confirm these findings, we examined MC3T3 osteoblast precursor cells incubated in sSema4D for prevention of differentiation by an alkaline phosphatase (AP) assay and loss of ability to produce matrix in a mineralization assay. We observed a decrease in AP activity (Fig 1*A*) and deposition of mineralized matrix (Fig 1*B*, Alizarin Red stain, *inset*) in MC3T3 growing under conditions favoring differentiation and mineralization when incubated with sSema4D, in a dose-dependent manner. Inhibition was dependent upon Sema4D binding to Plexin-B1 since this effect was lost in MC3T3 cells where Plexin-B1 was silenced with short hairpin (sh)RNA (Fig 1*C*, Plexin-B1 shRNA, *lower panel*, *second row*; knockdown confirmed by immunoblot, *top panel*), or where cells were incubated with an anti-Sema4D blocking antibody (Fig 1*C*, *lower panel*, *last row*), an antibody that our group and others have shown to block interaction with Plexin-B1 and inhibit Sema4D functioning in angiogenesis, tumor growth, and immunomodulation [28–30]. The results of the mineralization assay are quantified in the bar graph in Fig 1*D*. Taken together, these results confirm that Sema4D, acting through Plexin-B1, inhibits the ability of osteoblasts to form bone.

### Sema4D-mediated production of IL-8 by osteoblasts stimulates osteoclastogenesis

**Fig 2:**
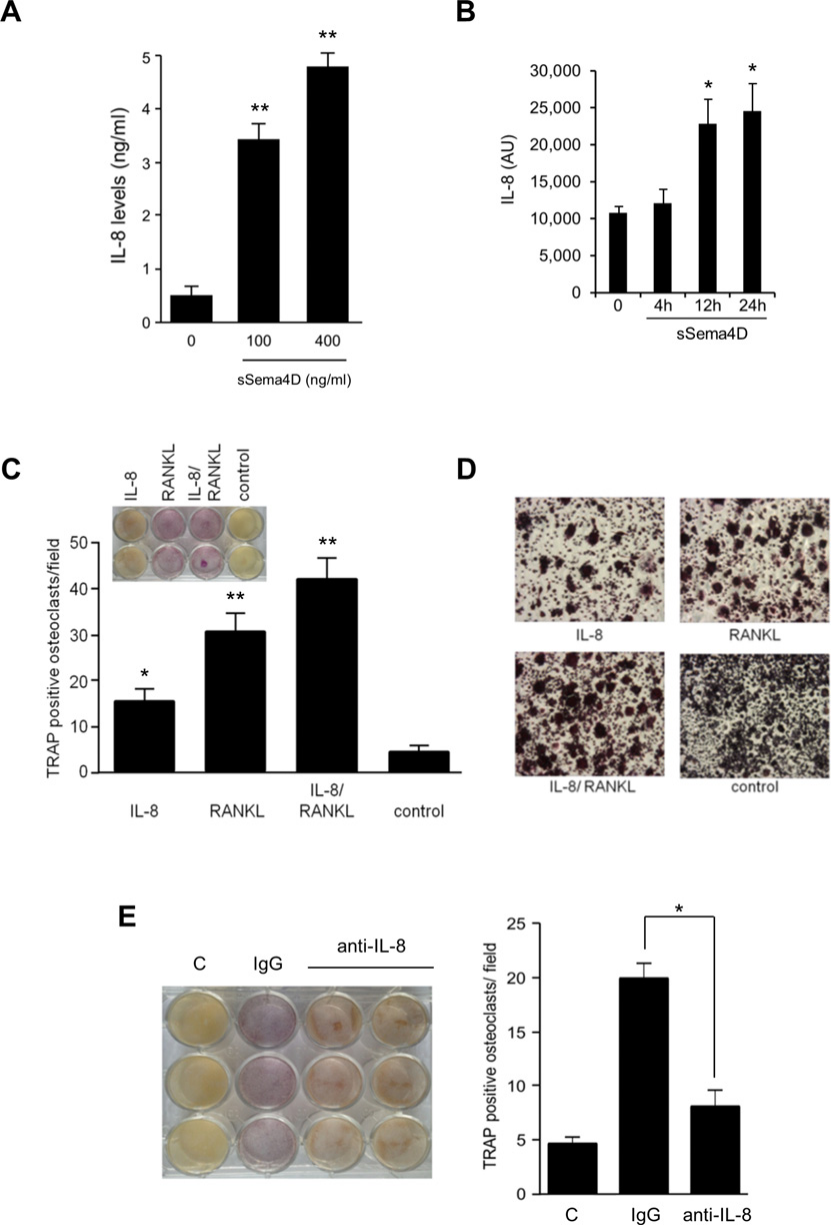
Sema4D stimulates production of IL-8 from osteoblasts, which promotes osteoclast differentiation. **A.** sSema4D treatment of the human osteoblast line HOB induces production of IL-8 in a dose-dependent manner (IL-8 concentration in ng/ml, Y-axis). **B.** An IL-8 ELISA performed on media conditioned by sSema4D treated HOB confirms this increase (Y-axis, arbitrary units (AU)) out to 24 hrs. **C.** IL-8 promotes osteoclastogenesis of RAW264.7 cells, particularly in the presence of RANK-L. TRAP assay is shown on top (inset) with the results quantified in the bar graph (average number of TRAP positive osteoclasts/ field, Y-axis). **D.** RAW264.7 cells are observed to fuse into multinucleated osteoclasts in the presence of IL-8. RANK-L enhances this response. **E.** Media conditioned by sSema4D treated osteoblasts induces osteoclastogenesis of RAW264.7 cells, a response lost in the presence of IL-8 blocking antibody, demonstrating the importance of this cytokine in differentiation. C, RAW264.7 treated with media conditioned by control osteoblasts (vehicle control), first column. Isotype matched IgG, second column. Anti-IL8 antibody, third and fourth columns. TRAP assay, left; Quantification, right (*, p ≤ 0.05; **, p ≤ 0.01).

Many factors mediate communication between osteoclasts and osteoblasts to balance bone resorption and formation. We further examined the effects of Sema4D on osteoblast-osteoclast communication by looking for changes in levels of well-known mediators of bone homeostasis by ELISA. The human osteoblast cell line HOB failed to show any changes in RANK-L or OPG when treated with sSema4D (*data not shown*) but we did note a robust increase in IL-8 production occurring in a dose-dependent manner (Fig 2*A*). These results were confirmed over a time course in an IL-8 ELISA (Fig 2*B*). We have previously linked Sema4D/ Plexin-B1 signaling to generation of IL-8 (in endothelial cells) [10] and IL-8 is believed to stimulate osteoclast activity [11]. Indeed, Sema4D has been linked with the promotion of osteoclastogenesis and pathological bone resorption [31]. To determine if IL-8 could drive differentiation of the monocyte cell line RAW 264.7 towards osteoclasts, we looked for tartrate-resistant acid phosphatase (TRAP) positivity, an indicator of osteoclast differentiation, in cells grown with IL-8, and determined their ability to fuse into large multinucleated mature osteoclasts *in vitro*. Results indicate that IL-8 does, in fact, promote RAW 264.7 differentiation (Fig 2*C*) and fusion into mature, multi-nucleated cells (Fig 2*D*), an effect independent of, but augmented by the addition of RANK-L. This could be prevented by co-incubation with anti-IL-8 antibody (Fig 2*E*, TRAP quantification shown in the bar graph, *right panel*). These results show that in addition to inhibiting osteoblasts, Sema4D can also simultaneously stimulate osteoclasts through IL-8, conditions strongly favoring bone resorption.

### Sema4D induces production of LIX/CXCL5 by osteoblasts, which stimulates osteoclastogenesis

**Fig 3:**
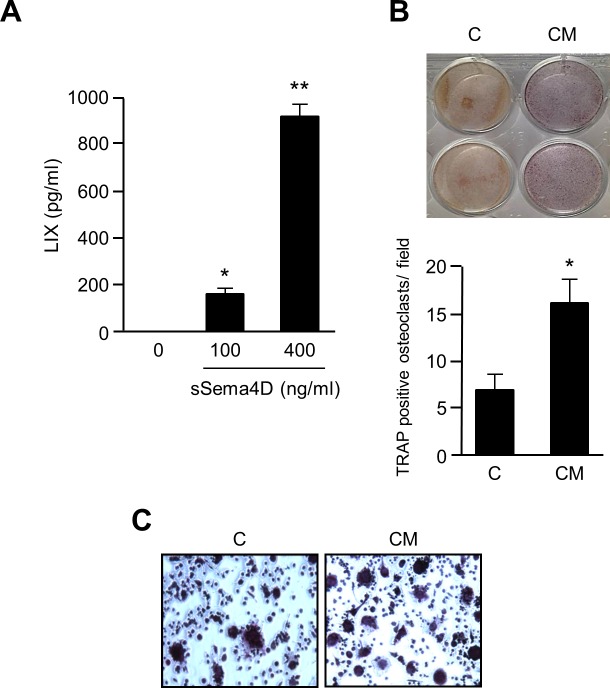
LIX/CXCL5, the murine homologue of IL-8, is produced by MC3T3 in the presence of sSema4D and promotes osteoclastogenesis. **A.** An ELISA performed on MC3T3 revealed increasing concentrations of LIX/CXCL5 (LIX, pg/ml, Y-axis) in increasing concentrations of sSema4D. **B.** Media conditioned by sSema4D treated MC3T3 (CM) induces osteoclastogenesis of RAW264.7 cells, compared to controls (C). TRAP assay, top; Quantification of results, bottom (average number of TRAP positive osteoclasts/ field, Y-axis; *, p ≤ 0.05; **, p ≤ 0.01). **C.** RAW264.7 cells are observed to fuse into multinucleated osteoclasts in the presence of media conditioned by sSema4D treated MC3T3.

LIX/CXCL5 is the murine homologue of human IL-8 [32]. To determine if Sema4D could induce production of this factor from murine osteoblasts, and to measure the effects on osteoclasts, we grew MC3T3 cells in increasing concentrations of sSema4D. An ELISA for LIX/CXCL5 detected increasing concentrations of this protein in conditioned media (Fig 3*A*), similar to the results observed in HOB for IL-8. A TRAP assay using media conditioned by sSema4D treated cells demonstrated increased positivity compared to controls (Fig 3*B*) and a greater number of large, multinucleated osteoclasts in culture (Fig 3*C*). Taken together, these results suggest that Sema4D-induced LIX/CXCL5 has osteoclastogenic effects in mice.

### Breast cancer cell lines produce Sema4D, which inhibits bone deposition and promotes RhoA-dependent IL-8 production by osteoblasts

**Fig 4:**
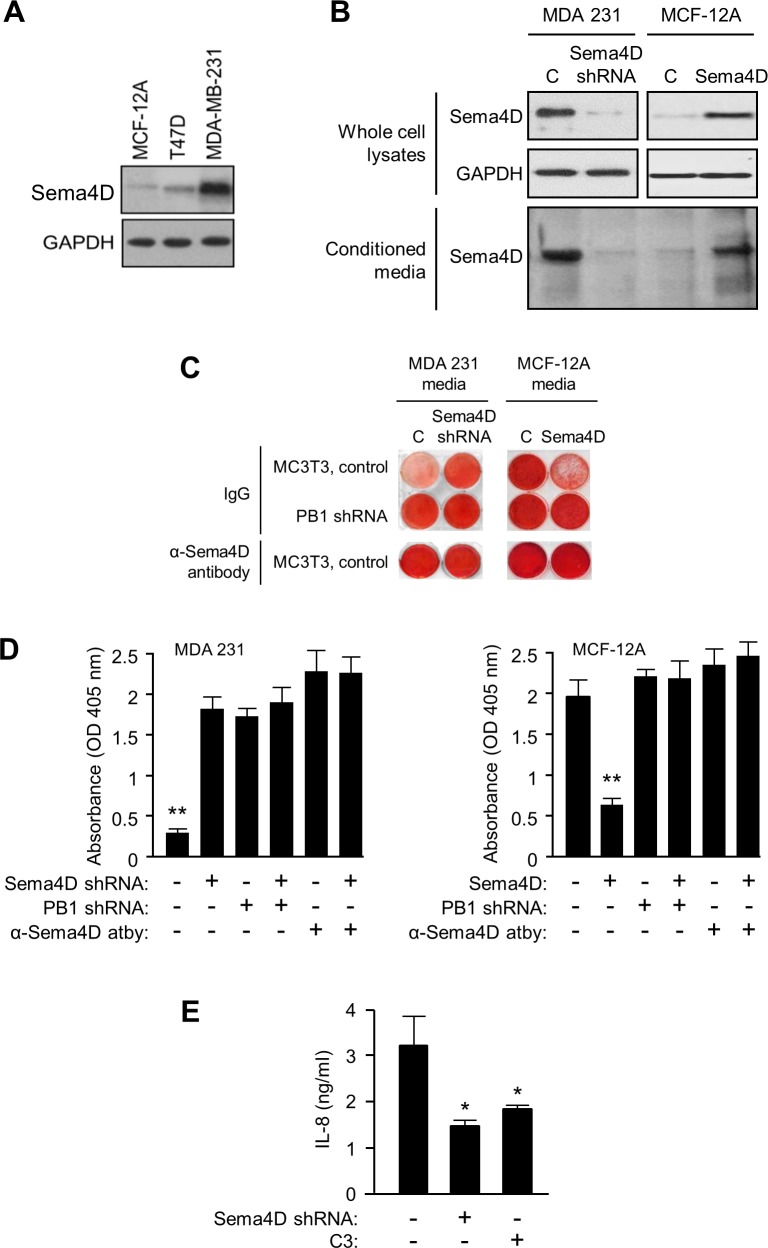
Sema4D produced by breast cancer cells inhibits mineralization and promotes RhoA-mediated production of IL-8 by osteoblasts. **A.** Breast cancer cell line MDA-MB-231 expresses high levels of Sema4D in an immunoblot compared to the non-tumorigenic line MCF-12 and T47D, which rarely metastasize to bone (top panel). GAPDH was used as the loading control (lower panel). **B.** MDA-MB-231 cells infected with lentivirus coding for a scrambled shRNA (C) express Sema4D in whole cell lysates, levels of which are greatly reduced in cells infected with lentivirus expressing Sema4D shRNA (top left panel). MCF-12A cells, which express very little endogenous Sema4D (C), are induced to overexpress it through lentiviral-mediated transfer of the wild-type construct (Sema4D, top right panel). GAPDH was used as a loading control (middle panel). sSema4D is present in media conditioned by control MDA-MB-231 cells and MCF-12A overexpressing Sema4D, but not in MDA-MB-231 with silenced Sema4D or control MCF-12A infected with a lentivirus containing an empty vector (bottom panel). **C.** MC3T3-mediated matrix deposition and mineralization, shown by Alizarin Red stain, is inhibited by media conditioned by control MDA-MB-231 (C, first column, left panels), but not in those where Sema4D is silenced by shRNA (Sema4D shRNA, second column, left). When Plexin-B1 is silenced in MC3T3 (PB1 shRNA, second row), or anti-Sema4D blocking antibody is administered (bottom row), mineralization is restored. Media conditioned by control MCF-12A had no effect on mineralization (C, first column, right panels) unless these cells were over-expressing Sema4D, in which case there was inhibition (Sema4D, second column, right). Mineralization was restored when Plexin-B1 was silenced in osteoblasts (PB1 shRNA, second row) or blocking antibody was present (bottom row). Isotype matched IgG was used as a control for both experimental groups. **D.** Quantification of results by absorbance at 405 nm (Y-axis) of the results shown in (B) from three independent experiments. MDA-MB-231 cells, left bar graph; MCF-12A, right. **E.** Incubation of the human osteoblast line HOB with media conditioned by MDA-MB-231 induces production of IL-8 (concentration in ng/ml, Y-axis), unless Sema4D is silenced in MDA-MB-231 by shRNA or Plexin-B1 signaling to RhoA is inhibited by addition of the *Clostridium botulinum* toxin C3 (*, p ≤ 0.05; **, p ≤ 0.01).

We have previously shown that breast cancers overexpress Sema4D [7]. These results were also seen in an immunoblot of a panel of breast cancer lines relative to their ability to spread to bone in experimental models. MDA-MB-231 cells, a human breast cancer cell line that forms osteolytic bone metastases when inoculated into the arterial circulation of mice [33], expresses high levels of Sema4D relative to MCF-12A or T47D cells, lines which do not spread to bone (Fig 4*A*). To determine if Sema4D from breast cancer cell lines can affect osteoblast-mediated mineralization, we needed to alter production of this protein. MDA-MB-231 cells infected with lentivirus coding for a scrambled shRNA construct produce high levels of endogenous Sema4D in whole cell lysates (Fig 4*B*, *top left panel*, *first lane*, *C*), which we could silence in cells infected with lentivirus expressing shRNA for Sema4D (Sema4D shRNA, *second lane*). MCF-12A cells, which express very little endogenous Sema4D, were induced to overexpress it through lentiviral-mediated transfer of the wild-type construct (Fig 4*B*, *top right panel*). We have previously shown that Sema4D is secreted or shed into the media in cancer cells expressing it through the action of a protease [15]. To confirm this in our breast cancer lines, we analyzed conditioned media by silver stain of an SDS-PAGE gel. We observe sSema4D in media conditioned by control MDA-MB-231 cells and MCF-12A overexpressing Sema4D, but not in MDA-MB-231 with silenced Sema4D or control infected MCF-12A (Fig 4*B*, *bottom panel*). We then grew MC3T3, controls or cells where Plexin-B1 was silenced with shRNA, in media conditioned by these populations of MDA-MB-231 and MCF-12A cells, with or without the anti-Sema4D blocking antibody. MDA-MB-231 media suppresses MC3T3-mediated mineralization, unless Sema4D is silenced in these cells, anti-Sema4D antibody is present, or Plexin-B1 is silenced in MC3T3 and they can no longer respond to this protein (Fig 4*C*, *left panels*). Conversely, media conditioned by MCF-12A had no effect on mineralization unless these cells were over-expressing Sema4D, in which case there was inhibition unless Plexin-B1 was silenced in the osteoblasts or blocking antibody was present (Fig 4*C*, *right panels*). These results are quantified in the bar graphs in Fig 4*D*.

It has been shown that osteoblasts from Plexin-B1^−/−^ mice exhibit less GTP-bound, active RhoA than controls [6]. Our group and others have also shown that ligation of Plexin-B1 by Sema4D activates RhoA to initiate signaling [3,34]. To investigate mechanism and to determine the effects of breast cancer-derived Sema4D on production of IL-8 by osteoblasts, we grew HOB in media conditioned by control MDA-MB-231 or cells with silenced Sema4D, with and without the *Clostridium botulinum* toxin C3, which inhibits activation of Rho signaling pathways, and examined IL-8 production by ELISA. Significant IL-8 production was detected from cells growing in media conditioned by MDA-MB-231, which was lost where Sema4D was silenced or Plexin-B1 signaling inhibited in HOB by C3 (Fig 4*E*). Taken together, these results suggest that Sema4D from breast cancers can inhibit mineralization by osteoblasts and induce their production of the osteoclast stimulating factor IL-8 in a RhoA-dependent manner.

### Sema4D production by breast cancers promotes lytic skeletal metastasis

**Fig 5:**
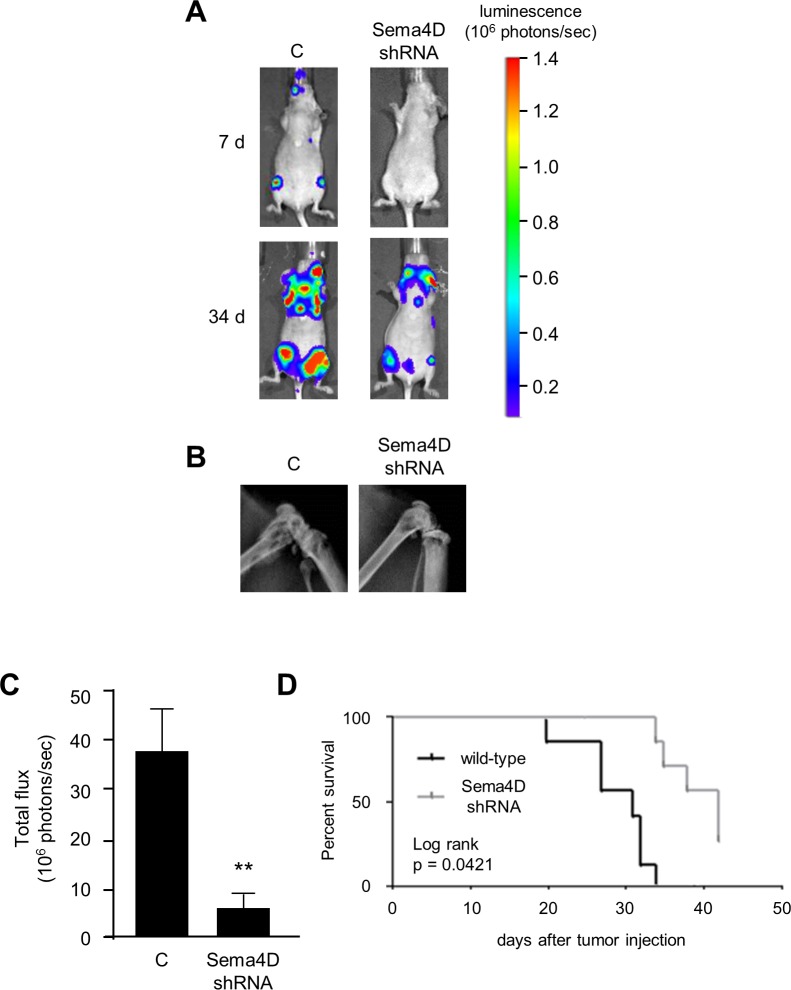
Xenografts with silenced Sema4D exhibit fewer and smaller lesions and longer survival compared to controls in a mouse model of skeletal metastasis. **A.** MDA-MB-231 expressing luciferase, controls (C) and with silenced Sema4D (Sema4D shRNA), were introduced into the systemic circulation of immunocompromised mice and developing lesions tracked by BLI. Representative lesions developing after 7 days (top row) and 34 days (bottom row) are shown. Luminescence, related to the number of collected photons, is shown on the right. **B.** Radiographs of representative lesions are shown. **C.** Mice grafted with MDA-MB-231 with silenced Sema4D exhibited fewer lesions (tumor burden, 10^6^ photons/sec, Y-axis) compared to controls (**, p ≤ 0.01). **D.** Kaplan–Meier curve reveals that mice grafted with MDA-MB-231 with silenced Sema4D exhibited longer survival compared to controls.

To determine *in vivo* biological significance of Sema4D expression in breast cancer as it relates to skeletal metastasis, we injected MDA-MB-231 cells stably expressing luciferase, controls and those with silenced Sema4D, into the left ventricle of immunosuppressed (nude) mice, which avoids passage through the lung, and tracked the development of skeletal lesions through bioluminescence imaging. Skeletal metastasis concentrated in areas of high bone turnover such as around the teeth and in the long bones, as previously reported [21]. We detected fewer and slower growing lesions from cells where Sema4D had been silenced, as shown by photon emission in Fig 5*A*. Radiographs also demonstrated less extensive lesions by MDA-MB-231 cells with silenced Sema4D compared to controls (Fig 5*B*). These results were confirmed when averaging total photon emissions of all luciferase-expressing lesions, which demonstrated a greater tumor burden in controls compared to MDA-MB-231 with silenced Sema4D (Fig 5*C*). Mice injected with tumor cell lines that failed to express Sema4D also exhibited an improved long term survival compared to controls over the course of the experiment (Fig 5*D*).

**Fig 6:**
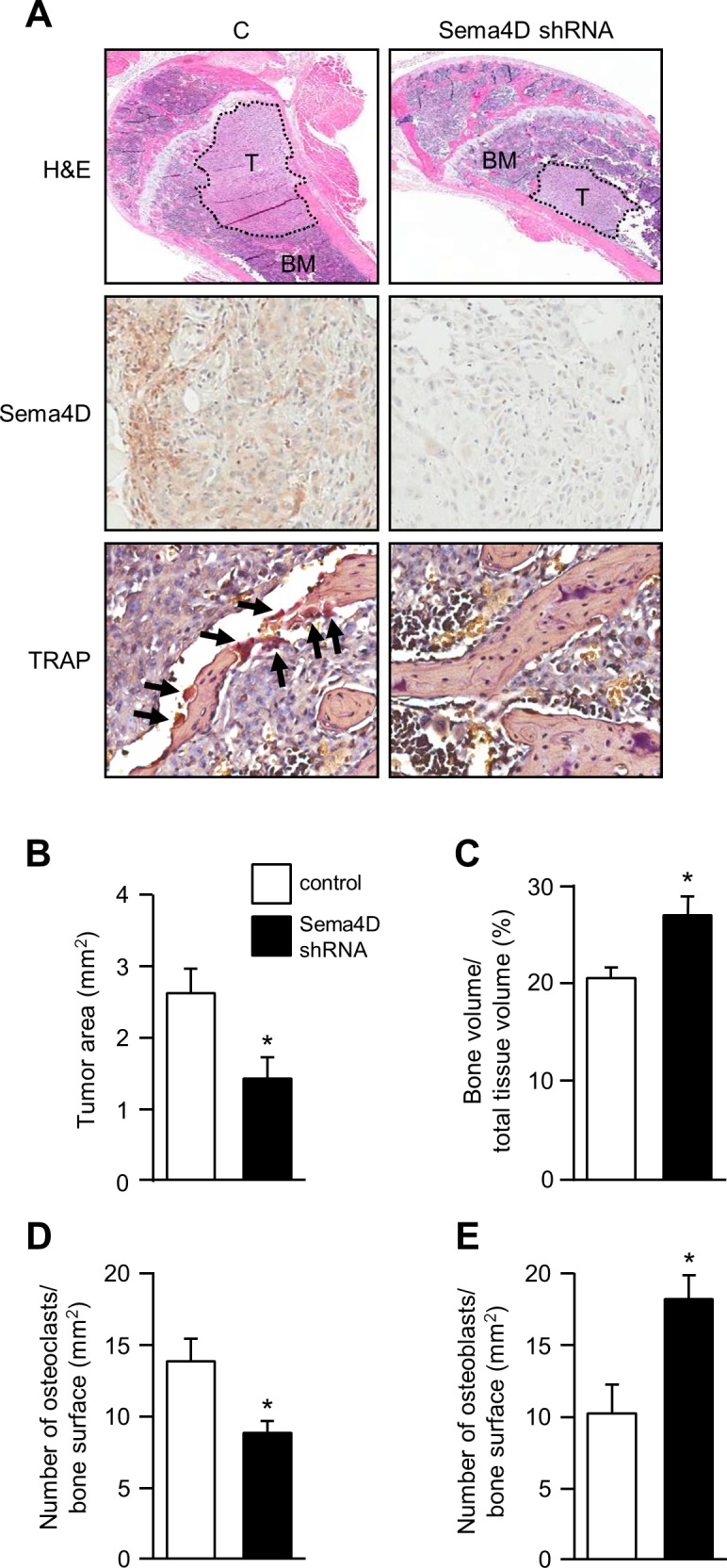
Sema4D promotes larger metastatic lesions, with fewer osteoblasts and more osteoclasts. **A.** Hematoxylin and eosin (H&E) stained sections of control (C) tumors or those with silenced Sema4D (Sema4D shRNA) metastatic to the femur (top row; T, tumor; BM, bone marrow; original magnification 10x). Control tumors on average were larger than those from cells where Sema4D was silenced. Sema4D silencing was confirmed by immunohistochemistry in tumor tissues (second row; original magnification 40x). Osteoclasts attached to bone in the tumor microenvironment were identified by TRAP immunohistochemistry (TRAP, bottom row, positive cells indicated by arrows; original magnification 40x). **B.** Histomorphometric analysis for total tumor area in formalin fixed paraffin embedded specimens confirmed that control tumors were larger (tumor area in mm^2^, Y-axis). **C.** Tumors made up of cells with silenced Sema4D exhibited greater preservation of bone volume (bone volume expressed as a percentage of total tissue volume, Y-axis). **D.** The number of osteoclasts present in lesional bone, as determined by TRAP positivity, was greater in control tumors (number of osteoclasts per mm^2^ of bone surface, Y-axis). (E) The number of osteoblasts in lesional bone was higher in tumors where Sema4D was silenced (number of osteoblasts per mm^2^ of bone surface, Y-axis; *, p ≤ 0.05).

We then examined skeletal metastases by immunohistochemistry and bone histomorphometric analysis for tumor size and the presence of osteoclasts and osteoblasts. Control MDA-MB-231 generated larger metastatic lesions (Fig 6*A*, H&E stain, *upper left panel*) compared to those expressing Sema4D shRNA (*upper right*; BM: bone marrow; T: tumor). Sema4D silencing was confirmed by immunohistochemistry (Fig 6*A*, *middle panels*). Immunohistochemistry for TRAP demonstrated a greater number of osteoclasts in control lesions (Fig 6*A*, *bottom left panel*, *arrows*) compared to those with silenced Sema4D (*bottom right*). Using morphometric analysis of femoral lesions [35], we confirmed that control tumors were larger (Fig 6*B*), while tumors made up of cells with silenced Sema4D exhibited greater preservation of bone volume (Fig 6*C*). The number of osteoclasts was also greater, on average, in metastases from control MDA-MB-231 cells (Fig 6*D*), while the number of osteoblasts was decreased (Fig 6*E*). Taken together, these results demonstrate that Sema4D production by tumors results in an increased number of osteoclasts, a reduction in osteoblasts, and subsequently larger skeletal lesions.

### Sema4D from breast cancer alters serum concentration of markers of bone homeostasis

**Fig 7:**
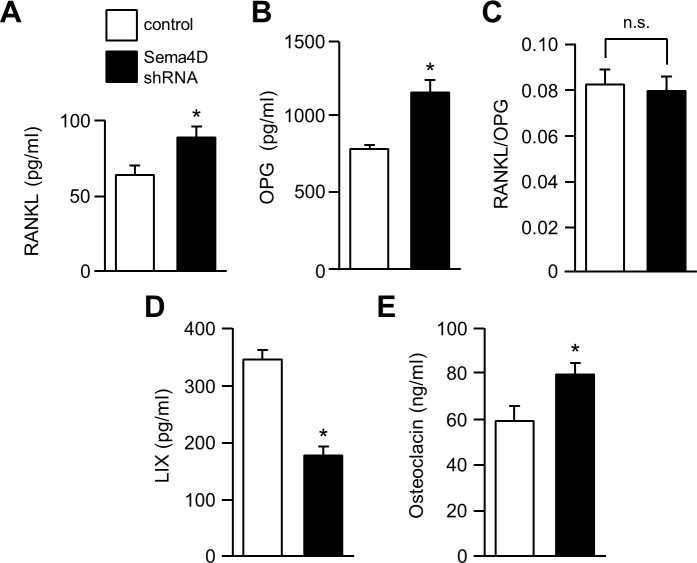
Silencing Sema4D in breast cancer changes serum levels of mediators of bone remodeling. **A.** ELISA performed for RANKL, an osteoblast-derived pro-osteoclastogenic factor, revealed higher levels in mice bearing tumors from MDA-MB-231 where Sema4D was silenced, suggestive of enhanced osteoblast activity, when compared to controls (RANKL levels in pg/ml, Y-axis). **B.** OPG levels are also higher in mice with tumors with silenced Sema4D (pg/ml, Y-axis). **C.** The ratio of the antagonists RANKL/OPG is not significantly different between the two populations, indicating the importance of other factors in the bone resorption observed in our model. **D.** In support of *in vitro* findings, mice bearing control tumors expressing Sema4D demonstrated significantly higher levels of serum LIX/CXCL5 (pg/ml, Y-axis). **E.** As expected, osteocalcin, a marker of osteoblast activity, is higher in the serum of Sema4D shRNA populations compared to controls (ng/ml, Y-axis; *, p ≤ 0.05; n.s., not significant).

To determine the effects of tumor Sema4D on bone physiology we tested the serum from experimental animals by ELISA for markers of bone homeostasis and turnover. Both RANKL (Fig 7*A*) and OPG (Fig 7*B*) were reduced in animals carrying tumors comprised of control MDA-MB-231, compared to tumors made up of cells with silenced Sema4D. Interestingly, there was no statistically significant difference between these two populations in the ratio of RANKL to OPG (Fig 7*C*), probably related to the fact that they are both products of osteoblasts and the relative levels between the two different populations might not change. This result also suggests that RANKL and OPG are not playing a significant role in the metastatic bone resorption measured in our system. Production of mouse LIX/CXCL5 was higher in controls producing Sema4D (Fig 7*D*). Like IL-8, this factor has been associated with bone resorption in mice [36]. The osteoblast marker osteocalcin was inhibited in controls (Fig 7*E*), consistent with the role of Sema4D in osteoblast suppression.

## Discussion

In recent years, new discoveries have expanded the role of plexins and semaphorins beyond that of nerve guidance and fasciculation and into the realm of bone metabolism. For example, Plexin-A1, which binds to class 3 semaphorins, has been implicated in control of bone density [37]. Indeed, it is now known that Semaphorin 3B promotes osteoclastogenesis and osteopenia in a mouse model [38] and that Semaphorin 3A exerts an osteoprotective effect by suppressing bone resorption and increasing bone formation [39]. The class 3 semaphorins have not been conclusively linked to regulation of skeletal metastasis in malignancy, but they are known to have inhibitory effects on breast cancer progression through their anti-angiogenic effects, which is lost in a subset of triple negative breast cancers (TNBC) and therefore might represent a potential target for therapy [40].

In normal bone maintenance, osteoblast differentiation needs to be suppressed until bone resorption is completed. This occurs in many ways, but Negishi-Koga, *et al*. demonstrated that osteoclasts suppress bone formation by producing Sema4D, which acts through its receptor Plexin-B1 on osteoblasts to activate the Sema4D–Plexin-B1–RhoA pathway [6]. It is believed that this suppresses osteoblast differentiation by attenuating IGF-I signaling [41] while simultaneously limiting the ability of cells to signal through integrins and adhere, spread, and migrate, functions essential for proper maintenance of bone [42]. One obvious consequence of this discovery is the value of Sema4D inhibition in the arrest of osteopenia. Sema4D^-/-^ mice demonstrate a mild osteosclerotic phenotype, identical to Plexin-B1^−/−^ mice and mice expressing dominant-negative RhoA, so this signaling pathway could have therapeutic implications in diseases such as osteoporosis. As cancer researchers, we immediately recognized another implication of these findings for skeletal metastasis. Some malignancies exhibit tropism for bone, and even in early stages form lesions which are osteolytic in nature. Stimulation of osteoclasts or direct resorption of bone by cancer cells would seem to be the obvious mechanism for bone lysis, but inhibition of osteoblasts, along with their negative feedback on osteoclasts, would also offer a distinct advantage in establishing a lytic bone lesion.

We have previously shown that many cancers, particularly those of epithelial origin, overexpress Sema4D [7] and therefore wanted to examine the biological significance in bone metastasis. We chose to examine breast cancer, as many are known to exhibit lytic skeletal lesions, but multiple myeloma and some prostate cancers also follow a similar progression of disease. Here we confirm that Sema4D inhibits osteoblast differentiation and ability to form a mineralized matrix *in vitro* in a Plexin-B1-dependent manner. We have shown that much of Plexin-B1 signaling, including migration of endothelial cells and activation of AKT and ERK, are mediated by the small GTPase RhoA [43]. Upon further investigation, we observed that IL-8 production by osteoblasts was dependent upon Plexin-B1 signaling to RhoA, resulting in indirect activation of osteoclasts. While RhoA signals through the cytoskeleton to influence cell migration, adhesion, and stress fiber formation, it also regulates gene expression, which could be the mechanism of IL-8 activation. However, the exact pathways engaged by RhoA and the downstream effectors necessary for IL-8 production by osteoblasts remain to be elucidated.

We observed that silencing of Sema4D expression in breast cancer cells through shRNA resulted in smaller and fewer skeletal metastases over time and greater life expectancy in a mouse model compared to controls. It should be noted, however, that despite differences in mineralization between these two populations, including a reduction in osteoblasts and enhanced osteoclastogenesis in metastatic lesions where cells were expressing Sema4D, we cannot rule out effects of Sema4D as a generalized factor influencing tumor aggressiveness. Indeed, overexpression of Sema4D (along with Plexin-B1) is correlated with histological tumor type, TNM stage, and metastasis in prostate and colon cancers [44,45] and a worse prognosis in both soft tissue sarcomas [46] and osteosarcoma [47]. Our group and others have shown that Sema4D promotes angiogenesis [3,4], so restriction of blood vessel growth into the metastatic lesions also is one possible contributor to the observed reduction in growth. We did not specifically measure breast cancer cell proliferation, invasion or vascularity in our experiments in an attempt to avoid duplication of already published reports, but Sema4D (or loss thereof) could certainly influence progression of metastatic lesions through these mechanisms. Nevertheless, our findings could have implications in therapy for TNBC, which lack dependence upon estrogen receptor, progesterone receptor and HER2 signaling and are therefore difficult to treat. Sema4D has been shown to activate Erb-B2/ HER2 (through Plexin-B1) to promote breast cancer metastasis [48], and although TNBC would probably fail to respond similarly, it's blockade would be worth investigating in these malignancies as a supplemental treatment along with more conventional approaches, if not to block cancer growth then to inhibit skeletal metastasis.

We propose a model of lytic skeletal metastasis where breast cancer cells newly colonizing a site in bone produce Sema4D which inhibits osteoblasts and causes them to produce IL-8, promoting osteoclast function. We do not postulate a role for Sema4D/Plexin-B1 signaling in the ability of certain tumor cell types to home to bone, but once at the site the end result of such alteration in osteoblast-osteoclast communication would be a more favorable microenvironment in which to establish a metastatic niche. The BLI results and histomorphometric analysis tend to support this model, as they describe a profile of decreased osteoblast function, enhanced osteoclast activity and larger, more numerous lesions when Sema4D is expressed in tumor cells. The serum ELISAs also lend support to our model. RANKL and OPG, antagonists in the regulation of osteoclast activity and bone resorption and mainly products of osteoblasts, are both lower in the serum of mice bearing tumors comprised of control cells still capable of producing Sema4D. However, the ratio of RANKL to OPG would be expected to be different between the two populations if these factors were playing a major role in tumor-induced bone resorption. We failed to observe this, suggesting instead that other factors are more important. We believe that not only is Sema4D significant in this process through its ability to directly inhibit osteoblasts but that its induction of LIX/CXCL5, which was higher in the serum of mice with control tumors, offers further stimulation of bone resorption through support of osteoclastogenesis. LIX/CXCL5 previously has been implicated in bone resorption in a mouse model of periodontal disease [36].

Sema4D in these metastatic lesions could come from different sources. For example, Sema4D is produced by osteoclasts, and we have demonstrated its expression in primary breast cancers in a tumor tissue array [7], but lymphocytes present in the metastatic microenvironment would produce Sema4D as well. To determine the importance of cancer cell Sema4D on bone metastasis, we eventually would like to investigate its expression in biopsies of primary human breast cancers and tumors metastatic to bone to see if there is any correlation with establishment of lytic skeletal metastases, as there seems to be for the cell lines we studied *in vivo*. The same difficulties would apply to IL-8 with regards to its origins but also its mechanism of production, particularly the link between IL-8 and NF-κB activation by osteoblast Plexin-B1. Such an analysis would require genetic targeting of osteoblasts in mice order to abrogate responses to Sema4D or the ability to generate LIX/CXCL5. Unfortunately, although mice are available that express tamoxifen responsive Cre in osteoblasts (and odontoblasts), there do not exist mice with floxed LIX/CXCL5. Finally, IL-8 likely is not the only factor produced by osteoblasts upon exposure to Sema4D that may enhance the establishment of skeletal metastases, as Sema4D is known to induce other cytokines [49]. This could be investigated by an HOB cytokine array for production of other proteins that influence osteoblast and osteoclast functioning, such as TGFβ, BMP2, and RUNX2, a key regulator of bone differentiation that has also plays a role in cell cycle regulation and tumorigenesis.

Like many malignancies, the molecular pathogenesis of breast cancer is complex, and the standard of care is administration of radio- and/or chemotherapy to induce death in the rapidly growing cancer cells. An advantage of studying ways to suppress metastasis instead of attempting to kill cancer cells is that the aggressive treatments necessitated by advanced disease can fail due to patient intolerance and the generation of resistant cancer cell clones arising from genetically labile progenitors under the selective pressures of cytotoxicity. Though treatable in its early stages, breast cancers eventually acquire the ability to proliferate inappropriately, avoid natural defenses, and resist therapy, suggesting the urgent need for innovative targets and strategies that suppress growth and invasion via an outcome other than cell killing. While the semaphorins and the plexins are crucial for cell adhesion, migration, invasion and metastasis, they have never been shown to be oncogenic or required for cell survival. Therefore, approaches directed against these proteins would not demonstrate toxicity to tumor cells, a feature that would represent an enormous advance in the field.

In bone homing malignancies, bisphosphonates are used to suppress skeletal metastases and the resulting complications of bone pain, pathologic fractures, hypercalcemia and other skeletal-related events, but this form of treatment inhibits osteoclasts and arrests bone remodeling, leading to “frozen bone” skeletal fragility and bisphosphonate-induced osteonecrosis of the jaw. Further benefits of our studies are that targeting Sema4D for inhibition would be a viable alternative to bisphosphonates, since it would not inhibit osteoclasts nor restrict skeletal remodeling and therefore could elicit an improved therapeutic result without the debilitating side effects of jaw osteonecrosis. Toxicity towards normal tissues would also not be an issue, as research into B-family plexins suggests that while important in normal development and malignancy, they are redundant for homeostasis of mature tissues [50].

## Conclusions

The findings presented here demonstrate that Sema4D enhances skeletal metastasis and could represent a new therapeutic target in the suppression of skeletal metastases for a subset of bone homing malignancies such as breast cancer.
